# Photocatalytic biocidal effect of copper doped TiO_2_ nanotube coated surfaces under laminar flow, illuminated with UVA light on *Legionella pneumophila*

**DOI:** 10.1371/journal.pone.0227574

**Published:** 2020-01-15

**Authors:** Martina Oder, Tilen Koklič, Polona Umek, Rok Podlipec, Janez Štrancar, Martin Dobeic

**Affiliations:** 1 Department of Sanitary Engineering, University of Ljubljana, Faculty of Health Sciences, Ljubljana, Slovenia; 2 Laboratory of Biophysics, “Jožef Stefan” Institute, Ljubljana, Slovenia; 3 Helmholz Zentrum Dresden Rossendorf, Ion Beam Center, Dresden, Germany; 4 Institute of Food Safety Feed and Environment, University of Ljubljana, Veterinary Faculty, Ljubljana, Slovenia; University of Akron, UNITED STATES

## Abstract

*Legionella pneumophila* can cause a potentially fatal form of humane pneumonia (Legionnaires’ disease), which is most problematic in immunocompromised and in elderly people. *Legionella* species is present at low concentrations in soil, natural and artificial aquatic systems and is therefore constantly entering man-made water systems. The environment temperature for it’s ideal growth range is between 32 and 42°C, thus hot water pipes represent ideal environment for spread of *Legionella*. The bacteria are dormant below 20°C and do not survive above 60°C. The primary method used to control the risk from *Legionella* is therefore water temperature control. There are several other effective treatments to prevent growth of *Legionella* in water systems, however current disinfection methods can be applied only intermittently thus allowing *Legionella* to grow in between treatments. Here we present an alternative disinfection method based on antibacterial coatings with Cu-TiO_2_ nanotubes deposited on preformed surfaces. In the experiment the microbiocidal efficiency of submicron coatings on polystyrene to the bacterium of the genus *Legionella pneumophila* with a potential use in a water supply system was tested. The treatment thus constantly prevents growth of *Legionella pneumophila* in presence of water at room temperature. Here we show that 24-hour illumination with low power UVA light source (15 W/m^2^ UVA illumination) of copper doped TiO_2_ nanotube coated surfaces is effective in preventing growth of *Legionella pneumophila*. Microbiocidal effects of Cu-TiO_2_ nanotube coatings were dependent on the flow of the medium and the intensity of UV-A light. It was determined that tested submicron coatings have microbiocidal effects specially in a non-flow or low-flow conditions, as in higher flow rates, probably to a greater possibility of *Legionella pneumophila* sedimentation on the coated polystyrene surfaces, meanwhile no significant differences among bacteria reduction was noted regarding to non or low flow of medium.

## Introduction

Hospitals, hotels, schools and homes for elderly people are often faced with the contamination of water pipeline systems caused by opportunistic pathogenic microorganisms such as *Mycobacterium avium*, *Pseudomonas aeruginosa* and/or *Legionella pneumophila* (*L*. *pneumophila*). Among the pathogenic microorganisms that mostly occur in the water systems bacteria *L*. *pneumophila* takes a special place because it can easily spreads and contaminates the drinking water supply systems and consequently cause a very dangerous Pontiac fever and/or Legionary disease. Considering that this disease can be spread predominantly by the aspiration of an contaminated aerosol, showers, pipes, tubes, cooling towers, thermal springs, water heaters, as well as respiratory devices, or nasogastric tubes in hospitals are predominant places for *L*. *pneumophila* contaminations and consequent humane infections [1, 2, 3]. In the United States in hospitals as much as 50% of all water supply systems in the buildings and 12–70% of the water supply systems are contaminated [1, 4]. Völker et al. [3] reports that in Germany every fourth home for the elderly and every seventh sport facility was contaminated with *L*. *pneumophila* at least once. In Hungary the results of monitoring showed that the *L*. *pneumophila* was present in 92% of the sampled hospitals, 71% of the hot water systems in hotels, over 50% of schools and 35% of dwellings [5]. Recently with climate change and increase in precipitation, the incidence of Legionnaire disease is increasing [2].

Bacteria *L*. *pneumophila* is present predominantly in the anthropogenic aquatic habitats and the water distribution systems, especially in the hot water systems where temperatures do not exceed 63°C and the aqueous pH is 5,0–9,2 [1, 2, 6]. Beside the ability to form a biofilm, *L*. *pneumophila* has the characteristic of intracellular parasitism and reproduction within 14 phagocytic amoebic organisms (eg *Acanthamoeba castellanii*, *Vermamoeba*, *Hartmanella vermiformis*), two ciliate protozoa (*Cyclidium spp*. and *Tetrahymena pyriformis*) and one type of mucous mold (*Dictyostelium discoideum*) [1, 6, 7, 8]. This further confirms the resistance of *L*. *pneumophila* to the environmental factors, while these bacteria were found in the microbial communities that form biofilm and are extremely resistant to chlorine (up to 50 ppm). For these reasons, the use of cleansing agents and disinfectants are often ineffective. Thus, in the last time many alternative methods are being studied, such as proper control of microbiom, the precise use of disinfectants or the structure of the water supply systems materials. The experimental use of antimicrobial substances as coatings on surfaces of the water systems is increasingly being considered in the last time. The aim of the antibacterial coatings is to reduce the adherence and growth of biofilm on the pipelines surfaces, which is the one of the most important measures against the spread of *L*. *pneumophila* [1, 2, 9, 10]. In general, biofilms are complex microbial communities, with interstitial water channels which allows the transfer of oxygen and feeding material to bacterial cells located in the biofilm [6]. Therefore, biofilms can be formed as complex multicomponent communities of human pathogen microorganisms as *E*. *coli*, *P*. *fluorescens*, *V*. *cholerae and Salmonella spp*. Since *L*. *pneumophila* has the property of the secondary colonization, which means it can be bind to the surface of the biofilms formed by other bacteria (eg *Pseudomonas aeruginsa*), this feature further enhances the survival and spread of *L*. *pneumophila*, because the biofilms are torn and spread to a several different plumbing sites due to erosion and peeling. The tearing of biofilms is dependent on materials, speed and type of water flow. In the water systems turbulent water discharged the biofilm and intensify the spread of bacteria in comparison to laminar, or stagnant water which does not stimulate the spread, but allows the unobstructed growth of biofilm [6, 11, 12]

*L*. *pneumophila* bacteria is capable of surviving under high humidity conditions and a relatively large range of ambient temperature between 20°C—50°C; however, it survives even at temperatures below freezing, due its protection in the biofilm and amebas. In addition, its ability to survive is also associated with symbiotic and parasitic interactions with other microorganisms. Therefore, it is highly resistant on large forms of disinfection. Its survival and reproduction in plumbing systems is also facilitate to favourable conditions for the biofilm growth, as the temperature, tube material (roughness, corrosion, presence of copper and iron ions, organic carbon), nutrient residues, calcium, magnesium, zinc and manganese. Especially favourable is the long water retention, which is a characteristic for the blind parts of the water distribution systems and water reservoirs [3, 13,14, 15]. Due to the contamination of the water systems with *L*. *pneumophila* bacteria, various procedures of sanitisation are carried out, starting with prevention, reducing water turbidity, filtration, classical disinfection with chlorine, oxygenation, copper and silver ionization, UV light, and thus bacteria is binding to solid particles with thermal and chlorine shocks, ozone, etc. For this reason, in prediction the risks of contamination and to monitor the presence and number of bacteria, it is necessary to identify potentially contaminated environments with *L*. *pneumophila*. In this context, Risk Analysis at Critical Control Points (HACCP) and Failure mode and Effects analysis (FMEA) should be involved [3, 16, 17, 18].

It should be note that in plumbing almost 95% of all bacteria including *L*. *pneumophila* are located in the biofilms and only 5% are present in the aqueous phase [19]. Therefore necessary preventive measures are needed, which in particular include the maintenance of an adequate water temperature at >55°C, preferably 65°C with a sufficiently long exposure time, frequent rinsing of pipes, the correct choice of the pipe materials and the avoidance of the long distances. For example, distances between the boiler room and distant taps can be even several hundreds of meters [5, 20, 21]. Unfortunately, the use of preventive measures does not ensure the long-term eradication of *L*. *pneumophila*, which makes necessary to use the combination of several types of measures. Due to the physicochemical factors it is of utmost importance that the spread of *L*. *pneumophila* is limited by thermal control, which is to maintain sufficiently high-water temperature of water. But also this measure is not effective insofar as the pipeline systems are not in condition of the convective mixing of water. The convective mixing is only possible with a higher temperature difference in water in the vertical tubes (20–60°C). Thus if the hot water do not discharge on the most distal taps due the convective mixing and especially if, after heat shock, the temperature of hot water returns to room temperature again, the thermal control against *L*. *pneumophila* is not effective [22].

In addition, *L*. *pneumophila* has the ability to obtain the resistance to certain types of disinfectants. Different factors are important for the successful disinfection of *L*. *pneumophila* with several side effects, which can occur during the disinfection process. These are the choice of disinfectant, concentration and operation time and especially the type and roughness of the pipe materials. Among the different disinfectants, mainly the chlorine disinfectants are properly functioning on the bacterial and eukaryotic structure of the microbiome in the drinking water [23]. Chloramine (NH_2_Cl) is shown to be the most effective, even if it acts slower than the other chlorine preparations, but more successfully penetrates the biofilm [24]. Since chloramine is mainly used for the disinfection of the plumbing systems, it should be considered that it has negative impacts on the plumbing materials as the corrosion of plastic, metal and rubber materials. In addition, chloramine produces side degradation products such as carcinogenic nitrosamines. No less problematic is chlorine dioxide (ClO_2_), with the acute health impact on humans.

Regarding to this, alternative methods were investigated. Recently, for the prevention against the pipeline bacterial colonization, one of the methods is the treating of drinking water with copper or silver ions. This system is based on copper and silver ions releasing in an electrical chamber where the water is exposed to electric currents between two anodes. According to some studies, even a 6 log reduction of bacteria can be achieved when the pH of the water was between 7.0 and 9.0 [6, 12]. However, this method has also negative side effects as the toxicity of both elements and their negative organoleptic effects on drinking water. The aim of the water pipelines disinfection is also to destroy amoeba that are inherently pathogenic and potential carriers of *L*. *pneumophila*. It was found that the free *L*. *pneumophila* were more sensitive to chlorine and chlorine dioxide than the bacteria protected in amoebas [25]. Recently, it has been striving to control the hygiene of the water systems also by the probiotic approaches by deliberately inoculating beneficial microbes to create a specific microbiome that would limit or prevent the growth of opportunistic pathogens [8].

While *L*. *pneumophila* poses a risk of human infection when spread by aerosol, various air purification filters including HEPA, electrostatic air filters, ion air purifiers and air filters with bioactive nano-filters are implemented [26].

Due to a numerous only conditionally successful disinfection methods and their problematic residues effects as toxicity and residua, the use of nano-technological preparations on the surfaces of the plumbing materials has been tested during the last time. The antibacterial properties of magnetic colloids, enriched with silver nanoparticles, or in conjugation with stabilizers, or titanium dioxide, which are supposed to be microbiocidal on the target microorganisms, are studied. The results of these studies show a high antibacterial capacity against *L*. *pneumophila* [9]. Some other studies demonstrate that the application of nano-silica dioxide on the surfaces can possible create a hydrophobic layer, which can reduce the formation of biofilms and the adhesion of heterotrophic bacteria to the surfaces [27].

However also the use of nanomaterials is facing to a variety of problems, as the toxicity, application manner, loss of antimicrobial function (due to the load of macromolecules and dead microorganisms as the protective layers on the surfaces coated with nano coatings), sperability, roughness of materials surface, etc. In our previous investigations, we show that the copper-doped TiO_2_ nanotubes deposited on surfaces of different materials and illuminated with low power of UV-A can retard the growth of testing microorganism *Listeria innocua* by 80%. We concluded that the TiO_2_ nanotubes have a potential to be employ for continuous disinfection of surfaces [28, 29]. In view of the promising results of previous research, we further examined the effectiveness of nano materials in preventing the colonization of *L*. *pneumophila* in some simulated conditions as they arise in drinking water plumbing systems. Since we have shown previously that copper doped TiO_2_ nanotubes can form stable deposition on many different surfaces, including polystyrene, polyethylene, and aluminum oxide, resistant to washing at different pH conditions, without abrasion, but under extensive water flow [28], in this work we, therefore, focused whether copper doped TiO_2_ nanotube surface coatings exhibit antibacterial effect against *L*. *pneumophila* and how this effect depends on the speed of the water flow and the intensity of the applied biologically non active UV-A light. Previously we have shown that copper doped TiO_2_ nanotubes exhibit biocidal effect against different strains in standing water conditions, in this work we therefore were, focused whether copper doped TiO_2_ nanotube surface coatings exhibit antibacterial effect against *L*. *pneumophila* and how this effect depends on the speed of the water flow and the intensity of the applied biologically non active UV-A light [28].

## Materials and methods

### Synthesis and characterization of TiO_2_ and Cu-doped TiO_2_ nanotubes

Rutile TiO_2_ powder (Aldrich, Titanium (IV) oxide, rutile powder < 5mm) was dispersed in Erlenmeyer flask with KOH to obtain the stock solution of concentration 1 mg/ml. Media and culture materials were obtained from Gibco–Invitrogen Corporation (Carlsbad, California). TiO_2_ nanotubes (TiO_2_NTs) and Cu-doped TiO_2_ nanotubes (Cu-TiO_2_NTs) were prepared from a H_2_Ti_3_O_7_ nanotube material. Detailed procedures are described in Garvas et al. [30] and Koklič et al. [28], respectively. In brief, for the preparation of TiO_2_NTs 400 mg of the H_2_Ti_3_O_7_ nanotube material was calcined at 380°C for 10 h. In the case of Cu-TiO_2_NTs, 400 mg of the H_2_Ti_3_O_7_ nanotube material was dispersed in 100 mL of 0.5 mM solution of Cu^2+^(aq) (source of Cu^2+^ was CuSO_4_·5H_2_O (Riedel de Haen)) and stirred at room temperature for 3 h. Afterwards was the solid material separated from the solution by centrifugation, dried at 100°C overnight and finally heated in air at 400°C for 10 h. For the preparation of Cu-TiO_2_ nanotubes with lower concentration of copper procedure was the same except that 200 mg of H_2_Ti_3_O_7_ nanotube material was disperse in 50 mL of 0.05 mM solution of Cu^2+^.

**Fig 1:**
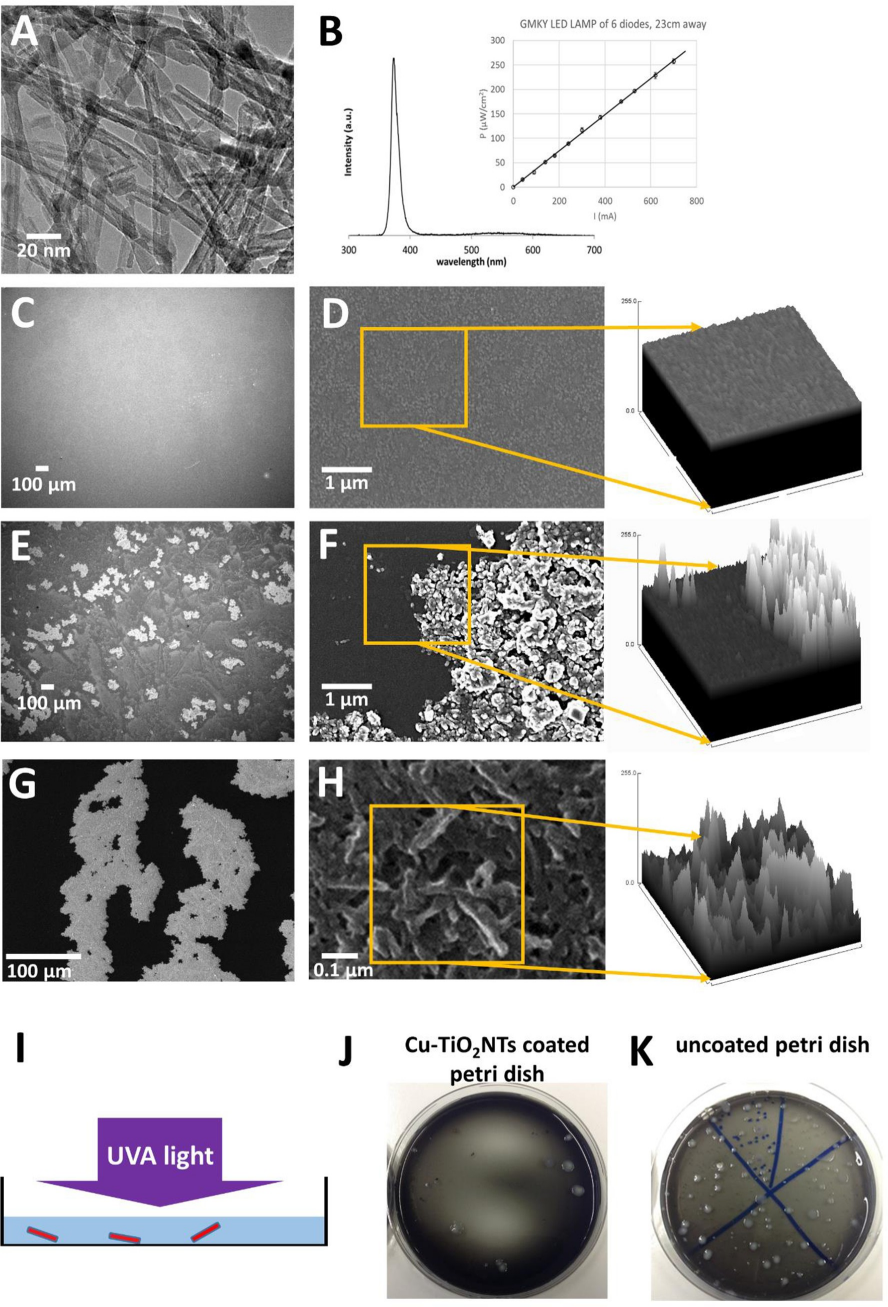
Copper doped titanium dioxide nanotube (Cu-TiO₂NT) coated surfaces. A) transmission electron microscopy images of Cu-TiO₂NTs. B) Emission spectrum of GMKY LED (U = 3.4 V, I = 280 mA, Popt = 70mW, Pel = 0.95W). Inset: intensity of LED lamp at the site of petri dish placement versus electrical current applied to the lamp. LED lamp consists of 6 GMKY diodes attached to 4 mm aluminum plate raised for 23 cm above illuminated surface.C, and D) Scanning electron microscopy (SEM) of polystyrene petri dish surface E, and F) SEM of Cu-TiO_2_NTs deposited on the petri dish G, and H) Helium ion microscopy of Cu-TiO_2_NTs deposited on the petri dish I) Scheme of the experimental setup. J) Typical result of survival of 100 *L*. *pneumophila* on a nanotube coated polystyrene dish in standing water with diluted growth medium in a petri dish illuminated with UVA light (15 W/m^2^) 24 hours in incubator at 36˚ C. Colonies of *L*. *pneumophila* can be observed as white spots. K) Colonies of *L*. *pneumophila* (white spots) on a control surface (bare polystyrene dish) at same conditions as in J.

Powder X-ray diffraction (XRD) patterns were acquired on a Bruker AXS D4 Endeavor diffractometer using Cu Ka radiation (1.5406 Å; in the 2*q* range from 10 to 70°). The morphology of the samples was investigated using a transmission electron microscope (TEM, Jeol 2100, 200 keV) (Fig 1A). Specimens for the TEM investigation were prepared by dispersing a powder sample ultrasonically in MeOH and depositing a droplet of the dispersion onto a lacy carbon-film supported by a copper grid. The copper content in Cu-TiO_2_NTs was determined using a field emission scanning electron microscope (SEM) equipped with an energy dispersive X-ray (EDX) elemental analysis system. Samples for EDX analysis were pressed into pellets and placed on a carbon tape on an Al sample holder. The holder with the pellet was coated with a thin carbon layer prior to the EDX analysis.

### Deposition of TiO_2_ and Cu-doped TiO_2_ nanotubes on polystyrene petri dishes

The deposition of either TiO_2_-NT or Cu-TiO_2_NTs was made on polystyrene (PS) petri dishes. The suspension of TiO_2_-based nanotubes with concentration of 1 mg/mL was processed with ultrasonic liquid processor (Sonicator 4000, Misonix) prior to the deposition on the petri dishes. Sonication was performed using 419 Microtip^TM^ probe, 15 min process time, 10 s pulse-ON time, 10 s pulse-OFF time and maximum amplitude (resulting in 52 W of power). Petri dishes were treated with compressed air 3 times for 3 s. and about 250 μL of the nanoparticle suspension was applied on each petri dish, immediately after compressed air treatment, and smeared evenly. After the deposition, the petri dishes were left in the oven on 60°C overnight. Then they were rinsed with water and put back in the oven at 60°C overnight.

### Polystyrene petri dishes surface roughness (Ra)

Surface roughness might affect the antibacterial effect of the surface. The bacteria adhered the most firmly to galvanized pipes while biofilm formation was the lowest to polypropylene pipes [15]. This is in agreement with the literature exploring the role of micro and nano scale surface roughness in controlling bacterial attachment onto surfaces, which has recently been an area of intense research [31]. To explore the role of the surface roughness in the antibacterial effect in the case of our polystyrene surface modifications, we compared the surface roughness of the control and nano-coated surface by using two different high-resolution imaging techniques for the analysis of coated and uncoated samples (Fig 1C and 1D). Scanning electron microscopy (SEM) was applied to study the spatial organization of larger nanoparticle aggregates on the petri dish surface (Fig 1E and 1F). In order to obtain even higher resolution images of the material surfaces samples were measured with Helium Ion Microscope (HIM) (Orion NanoFab, Zeiss) at Helmolz Zentrum Dresden Rossendorf (Fig 1G and 1H). HIM microscope equipped with GFIS trimer injection system can achieve few times higher lateral resolution compared to SEM which is 0.5 nm using 10–35 keV He ion beams. Main instrument parameters during the measurement of secondary electrons were 30 keV for ion acceleration which was performed in high vacuum with gun and chamber pressures of 3x10^-6^ and 3x10^-7^ mBar, respectively. Field of view of acquired images was tuned from 1mm down to 1 μm to perform nm resolution images of single nanoparticles. As a measure of surfaces roughness we plotted SEM and HIM images using “Surface Plot” function in ImageJ 1.52i [32].

### Preparation of bacterial inoculum and bacterial growth

Antimicrobial properties were tested on bacterium *Legionella pneumophila* subsp. *pneumophila* ATCC 33153 (*L*. *pneumophila*), obtained from the Czech Collection of Microorganisms (CCM, Brno, Czech Republic). The inoculum was prepared in a liquid medium Yeast Extract Broth with the addition of a *Legionella* BCYE α-growth supplement with cysteine 5 mg/L and incubated aerobically for 24 h at 36°C, in accordance with the ISO standard 11731:1999 [33]. This method was used in all experiments. After incubation the overnight culture contained approximately 10^6^ colony forming units (CFU) per millilitre (mL). Working suspensions with appropriate concentrations were achieved by several 10-fold dilutions.

### Biocidal effect of Cu-TiO_2_NTs coated petri dishes in no-flow (standing) conditions

**Fig 2:**
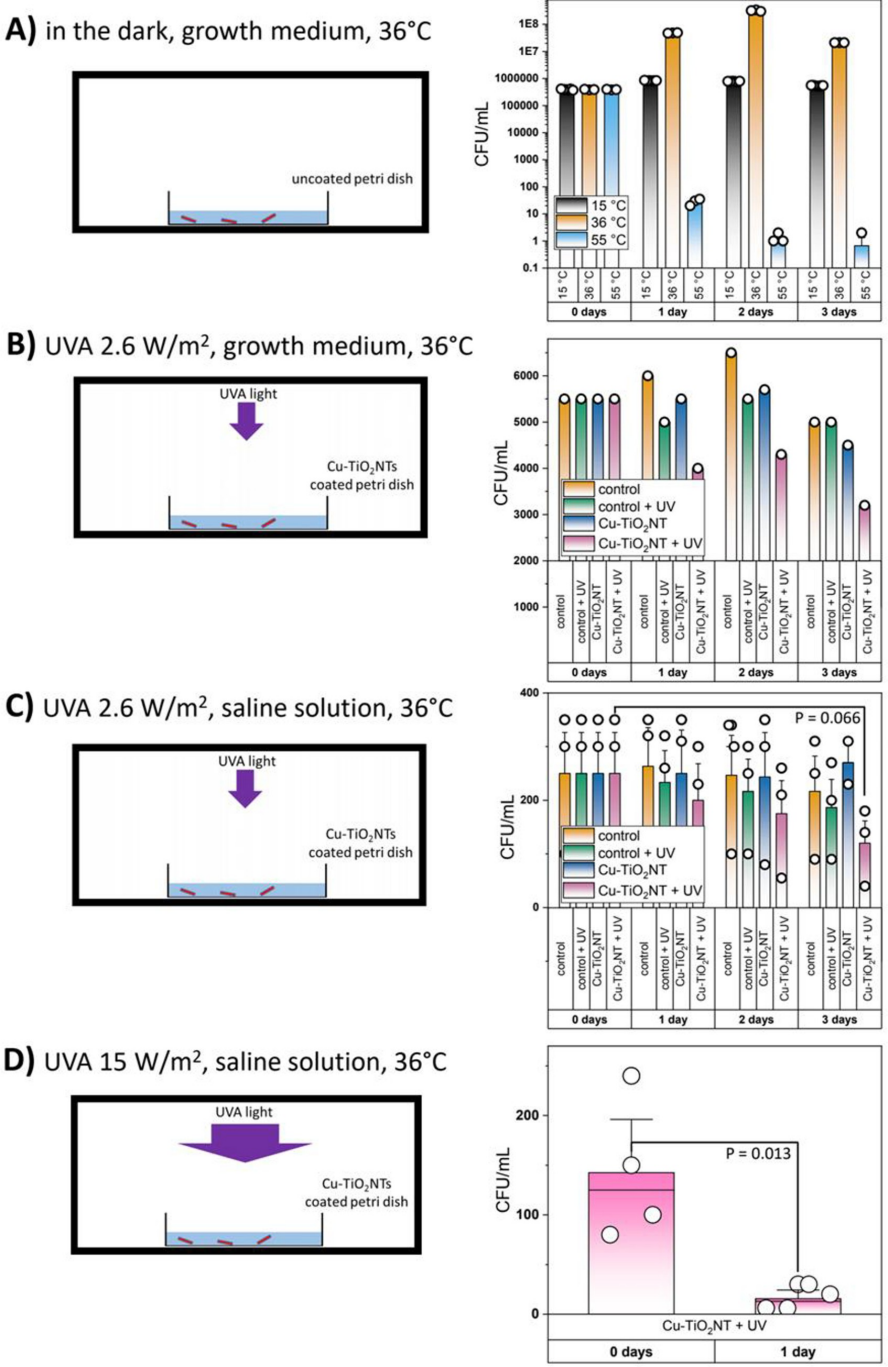
Biocidal effect of TiO_2_ nanotubes and copper doped TiO_2_ nanotubes coated surfaces in non-flow YEB growth medium and saline solution. Individual measurements of the number of colony forming units (CFU) are shown as open circles. Height of a bar represent mean value of CFU, error bars represent standard error. A) Growth curves of *L*. *pneumophila* in YEB medium in an incubator at three different temperatures: 15°C (black bars), 36°C (orange bars), and 55°C (blue bars) in uncoated petri dishes in absence of UV-A illumination. B) Survival of *L*. *pneumophila* in YEB medium with growth medium in a petri dish illuminated with low intensity UV-A light (2.6 W/m^2^) in an incubator at 36 ˚C on uncoated (control) petri dishes with (orange bars) and without (green bars) UV-A illumination and in petri dishes coated with copper doped TiO_2_ nanotubes without UVA illumination (blue bars) and with UV-A illumination (violet bars). The number of bacteria is reduced for about 55%, from 5500 CFU to 3000 CFU. C) Survival of *L*. *pneumophila* under same conditions as in B) except being in saline solution. Reduction in the number of bacteria is the greatest in petri dishes coated with copper doped TiO_2_ nanotubes illuminated with UVA light (violet bars) after 3 days of incubation. The number of CFU is reduced for about 50%, from 250±76 to 120±42 (two-tailed P-value = 0.066, two-tailed paired t-test: t = 3.702 with 2 degrees of freedom). D) Survival of *L*. *pneumophila* in saline solution in petri dishes coated with copper doped TiO_2_ nanotubes, illuminated with high intensity UVA light (15 W/m^2^) 24 hours in incubator at 36˚ C. Colonies are shown in Supplement (S6 Fig). Higher UVA light intensity reduces the number of CFU for approximately 90%, from 142±36 to 16±6 (two-tailed P-value = 0.013, two-tailed paired t-test: t = 3.513 with 6 degrees of freedom). Details of statistical analysis are shown in the Supplement.

In experiment we tested a biocidal effect of the Cu-TiO_2_NTs nanotube coated petri dishes on *L*. *pneumophila* in an incubator at 36 ˚C in no-flow conditions (Fig 2). In the first part of experiment the diluted growth medium Yeast Extract Broth (YEB) with addition of cysteine and 2 mL of bacterial suspension, with about 5000 CFU, was applied in each Cu-TiO_2_NTs coated petri dish (Fig 2B). In the second part of experiment only NaCl and 2 mL of bacterial suspension, with about 300 CFU/mL was applied in each Cu-TiO_2_NTs coated petri dish (Fig 2C and 2D). After 24 h of incubation, Buffered Charcoal Yeast Extract (BCYE) with the addition of cysteine was added in the petri dishes and incubated at 36°C for 24 hours in the incubator. For negative control non-coated petri dishes were used implementing the same procedure.

After the incubation, the colonies growing on the BCYE solid medium were counted and the results were expressed as CFU/mL. Experiment was repeated three times. During 72 h of incubation at 36°C in the aerobic conditions, the overnight culture with a concentration of 5.7 log CFU/mL was taken from the petri dishes every 24 hours.

### Antibacterial activity of Cu-TiO_2_NTs coated petri dishes under laminar flow

**Fig 3:**
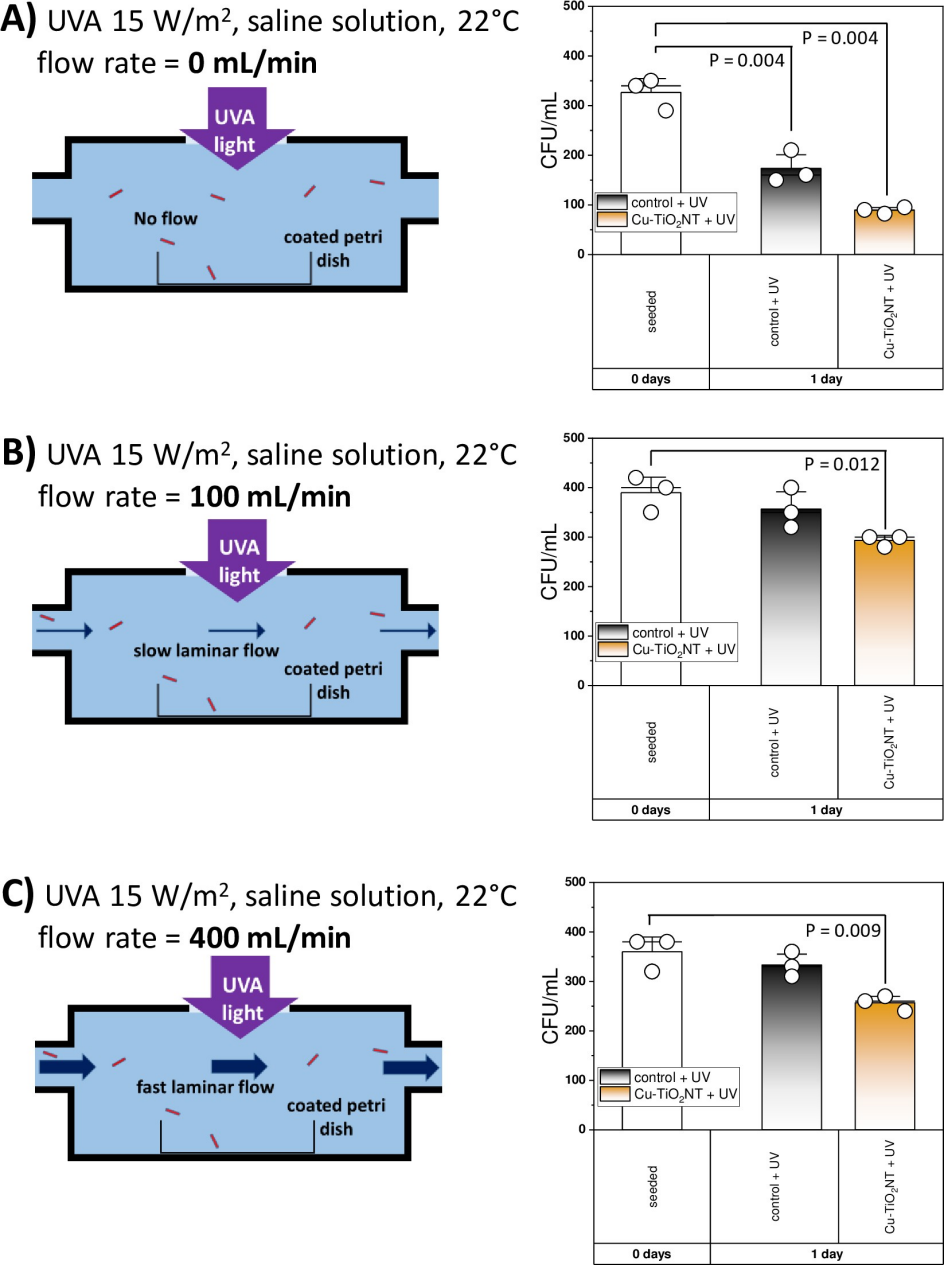
Survival of *L*. *pneumophila* in a laminar flow chamber in a petri dish illuminated with high intensity UVA light (15 W/m2). Volume of entire fluid was 2 L. Bacterium was grown overnight in suitable growth medium (overnight culture), which was diluted in a ratio of 1:30 into 2 L of the growth medium thus containing approximately 360 bacteria / mL (standard medium BCYE for *Legionella* with added cysteine). Diluted bacteria in saline solution were circulating for 24 hours at different rates of laminar flow. A) at 0 mL/min flow rate the number of bacteria is decreased for approximately 50%, from 327±19 to 173±18 (two-tailed P-value = 0.004, two-tailed t-test: t = 5.824 with 4 degrees of freedom) in uncoated petri dishes (black bars), and decreased for approximately 70%, from 327±19 to 93±5 (two-tailed P-value = 0.004, two-tailed t-test: t = 12.237 with 4 degrees of freedom) in Cu-TiO_2_NT coated petri dishes (orange bars); B) at 100 mL/min laminar flow rates the significant differences of approximately 25% CFU reduction in 1 day were observed only in copper doped TiO_2_ nanotube coated petri dishes (orange bars, two-tailed P-value = 0.012, two-tailed t-test: t = 4.422 with 4 degrees of freedom); C) at 400 mL/min laminar flow rates the significant differences of approximately 30% CFU reduction in 1 day were observed only in copper doped TiO_2_ nanotube coated petri dishes (orange bars, two-tailed P-value = 0.009, two-tailed t-test: t = 4.727 with 4 degrees of freedom). Details of statistical analysis are shown in the Supplement.

In part of experiments in the laminar flow conditions we tested the biocidal effect with the Cu-TiO_2_ nanotube coated petri dishes on *L*. *pneumophila* using a flow chamber with external dimensions of 110x400x50 mm. In flow conditions the bacterial suspension circulated at three different a constant flow rates of Q = 0 mL/min, Qmax = 100 mL/min and Qmax = 400 mL/min (Fig 3).

In the first part of experiments in a flow chamber we used 2 L of the the saline solution (0.9% NaCl) bacterial suspension, with the beginning concentration approximately 320 CFU/mL. Diluted bacteria were in flow chamber for 24 hours at room temperature 22±1 ˚C in no flow conditions (flow rate 0 mL/min) (Fig 3A).

In the second part of experiments in laminar flow conditions we used 2 L of the saline solution (0.9% NaCl) bacterial suspension, with the beginning concentration between 330 and 400 CFU/mL which was circulated at 22°C for 24 h. Saline as the circulating medium was used getting unsuitable conditions for the bacteria growth. Diluted bacteria were circulating for 24 hours at room temperature 22±1 ˚C in the laminar flow chamber at constant flow rate of 100 mL/min (first experiment) and then in the second experiment 400 mL/min (Fig 3B and 3C).

After all tests in the flow chamber, BCYE with the addition of cysteine was added in the petri dish and incubated at 36°C for 24 hours in the incubator. For the negative control non-coated petri dishes slides were used. After the incubation, the colonies growing on the BCYE solid medium were counted and the results were expressed as CFU/mL. Each of experiment was repeated three times.

In both experiments (no-flow and flow conditions) the samples were exposed to different intensities of a LED light source emitting the UV-A light (Fig 1B and 1I). The spectrum of emitted light was measured with a spectrometer (Ocean Optics USB2000+) and is shown in Fig 1B with peak wavelength 365 nm. The light source was made of high-power LED diodes (Shenzhen Hanhua Opto Co., Ltd.). The diodes were powered with electrical current source at U = 3.2 to 3.6 V, and maximum current 350 mA. We adjusted electrical current through the diodes to adjust intensity of emitted light. The intensity of emitted light versus electrical current was measured with a power monitor (Solo 2, Power & Energy Monitor, Gentec Electro-Optics, Inc., Quebec, QC, Canada). The curve showing linear dependence of emitted light intensity versus the electrical current is shown in the inset of the Fig 1B. The highest light intensity was achieved by placing a single GMKJ diode 4 cm above a petri dish. Exposures of 15 W/m^2^ UVA can be achieved economically with a single LED diode, dissipating 1W of total power, placed 4 cm above the petri dish.

## Results and discussion

### Phase composition, morphology, and location of copper doped TiO_2_ nanotubes and TiO_2_ nanotubes on polystyrene petri dishes

Firstly, both materials, Cu-TiO_2_NTs and TiO_2_NTs, were characterized by means of XRD to study their phase composition (S1 Fig). Both diffractograms are in agreement with the anatase structure (ICDD card no. 89–4203). In addition, no presence of CuO is observed in the copper loaded material (Cu-TiO_2_NTs) due to the low content of copper (S2 Fig). Morphology characterization of TiO_2_ nanotubes revealed that tubular structure of H_2_Ti_3_O_7_ material is preserved upon calcination (S3 and S4 Figs). Energy dispersive X-ray (EDX) analysis showed that the copper content in the material with higher amount of copper (Cu-TiO_2_NTs) is about 1.2 wt.%, and in the material with lower amount of copper is about 0.1 wt% (S2 Fig). Regarding the oxidation state of copper after calcination we may assume that it did not change as it was found by G. Li et al. [34] and Li et al. [35].To determine a local environment of Cu^2+^ in Cu-TiO_2_NTs we conducted an EPR (electron paramagnetic resonance) measurement of the sample with lower amount of copper (0.1 wt.%). From the recorded EPR spectrum (S5 Fig) it is evident that in the sample are at least two copper species in the oxidation state 2+. The component having g_||_ = 2.33 can be assigned to Cu^2+^ ions at substitutional cation sites in the TiO_2_ matrix while the component with g_||_ = 2.275 correspond to the Cu^2+^ in CuO clusters [36, 37]. After the deposition of the nanotubes onto smooth polystyrene petri dish surface (Fig 1C and 1D) the surface roughness increases (Fig 1E and 1F), however this didn’t decrease the antibacterial effect of the control surface (Fig 2C, orange bars vs blue bars), which is in agreement with the literature showing that bacteria can successfully colonise surfaces with an average surface roughness of the order of only a few nanometres or even less [38] and with our own published data showing that the adhesion of *L*. *pneumophila* was not the lowest on the smoothest material [15].

### Biocidal effect of Cu-TiO_2_NTs coated petri dishes in no-flow conditions

We tested biocidal effect of the CuTiO_2_NT coated petri dishes on *L*. *pneumophila* in no-flow conditions in the incubator at 36 ˚C (Figs 1J, 1K and 2). Fig 2A shows the growth curve of *L*. *pneumophila* at 15, 36 and 55 ˚C in the uncoated petri dishes in absence of the UV-A illumination. The number of *L*. *pneumophila* at 36 ˚C increased from the initial concentration (5.6 log CFU/mL) by about 3 log CFU/mL in the first 48 h. Their number after 72 h of incubation was 7.3 log CFU/mL.

Biocidal activity of Cu-TiO_2_ coated petri dishes, illuminated with the low intensity UV-A light (2.6 W/m^2^), decreases the number *L*. *pneumophila* in the growth medium (YEB) at 36 ˚C to about 55% within the 72 h of incubation (Fig 2B). We observed similar activity (50% reduction) of the Cu-TiO_2_NTs coated petri dishes also when *L*. *pneumophila* was incubated with the saline solution (Fig 2C). However, the photocatalytic disinfection method was much more efficient when we used higher intensity illumination (15 W/m^2^). In this trial the number of *L*. *pneumophila* decreased to about 90% in just 24 h of incubation (Fig 2D).

### Antibacterial activity of Cu-TiO_2_NTs coated petri dishes under laminar flow of saline solution

For experiment in the flow conditions we used a laminar flow chamber where the Cu-TiO_2_NTs coated petri dishes were placed inside, as shown schematically in Fig 3 and S8 Fig.

Before the experiment in flow conditions preliminary test of the biocidal effects on *L*. *pneumophila* in no-flow conditions was employed. Under no-flow conditions, the biocidal effect of nanotubes coated surfaces has been confirmed as biocidal activity of the Cu-TiO_2_NTs coated petri dishes in the saline solution at 22±1 ˚C in the no-flow conditions, illuminated with low intensity UV-A light (15 W/m2), demonstrated decreases of the number of *L*. *pneumophila* for 73% (P = 0.004) within the 24 h of incubation (Fig 3A).

We expected similar activity of the Cu-TiO_2_NT coated petri dishes also when *L*. *pneumophila* was incubated it saline solution 24 h, under the laminar flow conditions a constant flow rate of Qmax = 100 mL/min at 22 ˚C. But the results showed (Fig 3B), that the number of bacteria decrease on Cu-TiO_2_NT coated petri dishes for 25%, and on uncoated petri dishes for 8%.

The results at constant flow rate of Qmax = 400 mL/min in saline solution incubated 24 h, at 22 ˚C Fig 3C showed, that the number of bacteria decreased on Cu-TiO_2_NT coated petri dishes for about 30%. On uncoated petri dishes the number of bacteria decrease for 15%.

In an experiment in a laminar flow chamber polystyrene dishes were exposed to the low flow of medium. Significantly less viable bacteria (beginning concentration of *L*. *pneumophila* was 10^6^ CFU/mL) could be observed on the Cu-TiO_2_NTs coated in comparison to the non-coated petri dishes. Presumably the low flow of the medium did not affect the sedimentation of the bacteria onto the PS surfaces, and at the same time it prevents the accumulation of dead bacteria and the formation of a protective layer that would disable the contact between the Cu-TiO_2_NTs and the living bacteria from the flowing medium and thus their microbiocidal function. However higher microbiocidal efficiency of submicron coatings was shown through the higher intensity of the illumination with UV-A. Since the significant differences in higher reduction of bacterial counts on coated in comparison to uncoated polystyrene dishes occurred, this can be explained as contemporaneity of UV-A supporting efficiency to Cu-TiO_2_NTs coatings, not only on the function of UV-A itself. This means that higher UV-A illumination enhances the performance of the Cu-TiO_2_NTs coatings. In particular, microbiocidal activity of TiO_2_ nanotube coatings increases with the intensity of UV-A radiation, which stimulates the generation hydroxyl radicals through the mechanism explained in our previous work [28].

*Legionella* occurs naturally lakes and streams and can become a serious health concern when it grows in potable water system [37], such as kitchen and bathroom pipes and showers, with water flow rates ranging from 200 mL/min, in kitchen appliances, such as coffee machines, to 4 L/min in bathroom and kitchen faucets [39]. The flow rates we used are at the lower range of water flows of potable water, which we believe is relevant, since the risk for overgrowth of *Legionella* species is in domestic hot water systems, which are not in regular use, where water is standing for a prolonged period of time [40].

Considering that the water in the water supply systems of the non-industrial sector is predominantly standing than running, which is in favour of bactericidal effects of the Cu-TiO_2_NTs coatings, where bacteria has to come in close contact with the Cu-TiO_2_NTs coated surface. This is more readily achieved in standing water due to easier sedimentation of bacteria on the surfaces of the plumbing as compared to the water supply systems where water flow is higher.

We observed the highest antibacterial effect of nanotube coated polystyrene petri dishes in standing water (Fig 3A) when UV-A illumination of control surfaces was also effective. At higher flow rates, up to 0.4 L/min the UV-A treatment alone was not effective anymore, whereas the antibacterial effect on the nanotube coated surfaces was still significant. The flow rates we used are at the lower range of water flows of potable water, which we believe is relevant, since the risk for overgrowth of *Legionella* species is in domestic hot water systems, which are not in regular use, where water is standing for a prolonged period of time [40].

This effect is also evident also from our experiments where bacteria reduction was more than double in no flow conditions the (Fig 3A), as when polystyrene dishes coated with Cu-TiO_2_NTs were exposed to the circulation medium of saline solution in a laminar flow chamber at 100–400 ml/min at 22°C, with 15 W/m^2^ UV-A (Fig 3B and 3C). This is consistent with our previous results, where we have used small volumes of bacterial suspensions in order to facilitate close contact among bacteria and the nanotube coated surface [28]. In experiments presented here we used higher volumes of bacterial in order to simulate water supply systems, therefore the only parameter affecting the duration of the contact was therefore speed of the water flow. We have shown previously that copper doped TiO_2_ nanotubes produce more hydroxyl radicals than the undoped nanotubes, however the deposition of the nanotubes is nonhomogeneous (S7 Fig) and less copper doped TiO_2_ nanotubes can be stably deposited onto polystyrene surface [28], thus resulting in similar biocidal activity of both nanomaterial coated surfaces (S8 Fig). Thus, the key parameter determining the biocidal effect is the rate of the flow.

We believe that biocidal effect should be applicable also to other bacterial strains, *L*. *innocua*, *S*. *aureus*, *MRSA*, *E*. *coli ESBL*, and *E*. *coli*, as we have shown in our previous publication, albeit in standing water conditions and at 4°C [29]. We have also shown that the biocidal mechanism is based on the action of the hydroxyl radical (·OH), which is known to indiscriminantly affect any bacterial strain. Namely no enzyme is known with ability to catalyze the ·OH [41], which can indiscriminately oxidize organic matter, including DNA [42].

However, in this paper we, therefore, focused on a specific bacterium *L*. *pneumophila*, and on the water flow speed and light intensity of the light in order to show that antibacterial effect can be achieved at slow water flow speeds and at low light intensity with the wavelength almost in the visible range–UV-A light. Namely, *Legionella* occurs naturally lakes and streams and can become a health concern when it grows and spreads in human-made building water systems and potable water [37].

## Conclusions

The photocatalytic treatment with either copper doped TiO_2_ nanotubes (Cu-TiO_2_NT) or undoped TiO_2_ nanotubes (TiO_2_NT) deposited onto polystyrene surface can constantly prevent growth of a *L*. *pneumophila* in presence of YBE medium or saline solution at room temperature. The disinfection treatment presented in this experiment can be applied constantly using low intensity UV-A light. Thus, the results of experiment show the potential use of the Cu-TiO_2_NT coatings on polystyrene for inhibiting the growth of *L*. *pneumophila*. We have found that Cu-TiO_2_NT and TiO_2_NT coatings when irradiated with UV-A prevent the growth of bacteria both in the standing and in flowing medium. This is important from the view that *Legionella* reproduces and forms a biofilm predominantly in the still water. In this case, the contact of bacterial cells with coatings is prolonged. The experiment showed that the Cu-TiO_2_NT and TiO_2_NT coatings were microbiocidal at low, but in a certain proportion, also in higher flows, which is of great importance in terms of *L*. *pneumophila* water pipe-lines distribution, as this reduces the risk of bacterial spread downstream, without routine or sporadic heating of water above 55°C. Although the deposition of the nanotubes onto smooth polystyrene petri dish surface increases the surface roughness, this doesn’t affect the antibacterial effect of the surface when the surface is not illuminated with the UV-A light.

In this work we demonstrated the short term microbiocidal efficacy of UV-A illuminated TiO_2_ nanotube coatings on *L*. *pneumophila* indicating that such coatings could potentially be used in plumbing systems to maintain water uncontaminated. In the following research work, it will be necessary to study the durability and microbiocidal performance of these coatings over longer time periods and to investigate their effectiveness in preventing the growth of biofilms.

## Supporting information

S1 Fig XRD patterns of copper doped TiO_2_ nanotubes (Cu-TiO_2_NTs) and undoped TiO_2_ nanotubes (TiO_2_NTs). Only peaks corresponding to anatase phase (ICCD card no. 89–4203, ICDD stands for International center of difraction data database) are observed in both diffractograms.
(TIF)

S2 Fig **EDX spectra of copper doped TiO**_**2**_ **nanotubes (Cu-TiO**_**2**_**NTs, left) and B) undoped TiO**_**2**_ **nanotubes (TiO**_**2**_**NTs) (right).** The inset in figure A unambiguously shows presence of copper in Cu-TiO_2_NTs. The amount of copper determined from the EDX spectrum of Cu-TiO_2_NTs is 1.2 wt%. In both spectra are present signals of C and Al what is a consequence of the specimen preparation for EDX analysis (please see Materials and Methods in the main paper).
(TIF)

S3 Fig **Low (a) and (b) high magnification TEM images of TiO**_**2**_ **nanotubes (TiO**_**2**_**NTs) obtained by calcination of H**_**2**_**Ti**_**3**_**O**_**7**_ **nanotubes at 380°C for 10 h.** Nanotubes wall structure can be clearly seen in the inset of image (b). The average nanotube diameter is between 8 nm and 9 nm.
(TIF)

S4 Fig **TEM images of copper doped TiO**_**2**_ **nanotubes (Cu-TiO**_**2**_**NTs) obtained by calcination of copper doped H**_**2**_**Ti**_**3**_**O**_**7**_ **nanotubes at 400°C at low (a) and (b) high magnification.** Upon calcination nanotube morphology is retained.
(TIF)

S5 Fig EPR spectrum of Cu-TiO_2_NT sample containing 0.1 wt. % of copper recorded at 100 K. The black lines indicate hyperfine structure for two coexisting signals in Cu-TiO_2_NTs with 0.1 wt. % of copper. The EPR measurement was performed on an X-band EPR spectrometer Bruker ELEXYS, Type W3002180, using 10 Gauss (10^−3^ T) modulation amplitude, 100 kHz modulation frequency, 3 μW microwave power and 2000 G sweep.
(TIF)

S6 Fig **Survival of different number of *Legionella pneumophila*** (100 bacteria seeded: A, B; 1,000 bacteria seeded: C, D; 10,000 bacteria seeded: E, F) in saline solution in a petri dish coated with copper doped TiO_2_ nanotubes (column Cu-TiO_2_NTs + UV) illuminated with UVA light (15 W/m^2^) incubated for 24 hours in incubator at 36˚ C compared to control (column PS–bare polystyrene surface).
(TIF)

S7 Fig **SEM image Cu-TiO**_**2**_**NTs deposited on a petri dish (a) and EDX spectra (b,c).** Red frame in the SEM image corresponds to an area over which the spectrum (a) was taken, while blue frame corresponds to an area over which the spectrum (b) was taken. Spectrum (b) shows presence of Ti and O coming from TiO_2_, while in the spectrum (c) there are just signals of C and O. Presence of oxygen in this spectrum is probably a consequence of the cleaning process where compressed air treatment was used.
(TIF)

S8 Fig Survival of *Legionella pneumophila* in laminar flow chamber in a petri dish illuminated with high intensity UVA light (15 W/m^2^). Volume of entire fluid was 2 liters. *Legionella* was grown overnight in suitable growth medium (overnight culture), which was diluted in a ratio of 1:30 into 2 liters of growth medium thus containing approximately 360 bacteria / mL (standard medium BCYE for *Legionella* with added yeast extract). Diluted bacteria were circulating in saline solution for 24 hours in a laminar flow chamber at 22°C.
(TIF)

S1 Appendix (DOCX)
