# Supplementary figures and images for: Photocatalytic biocidal effect of copper doped TiO2 nanotube coated surfaces under laminar flow, illuminated with UVA light on Legionella pneumophila
Source: PLoS One. 2020 Jan 15;15(1):e0227574. doi: 10.1371/journal.pone.0227574 (PMC6961935; doi:10.1371/journal.pone.0227574)

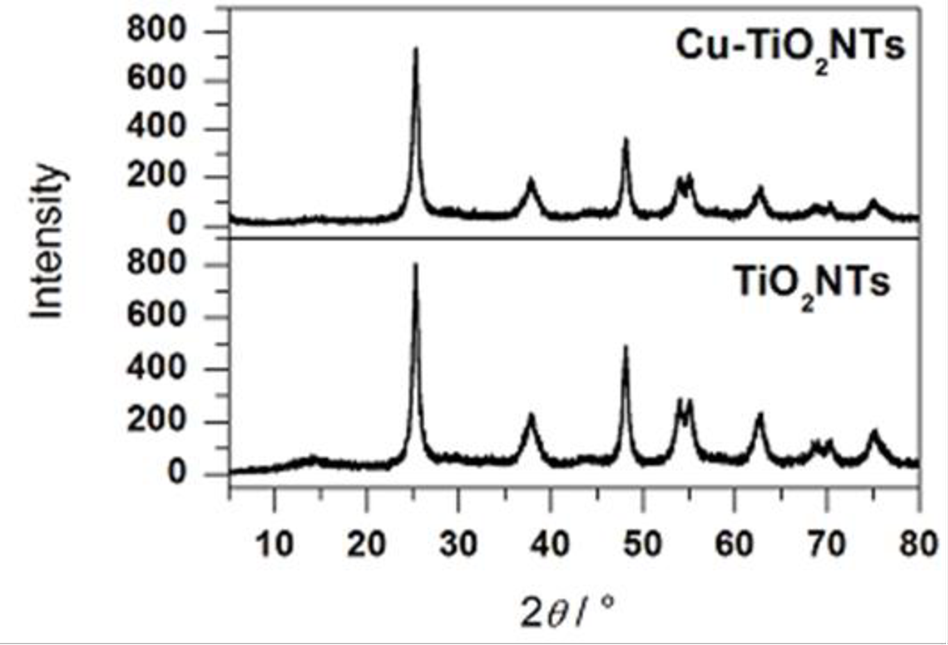

Supplement: S1 Fig — Only peaks corresponding to anatase phase (ICCD card no. 89–4203, ICDD stands for International center of difraction data database) are observed in both diffractograms. (TIF) [file pone.0227574.s001.tif]

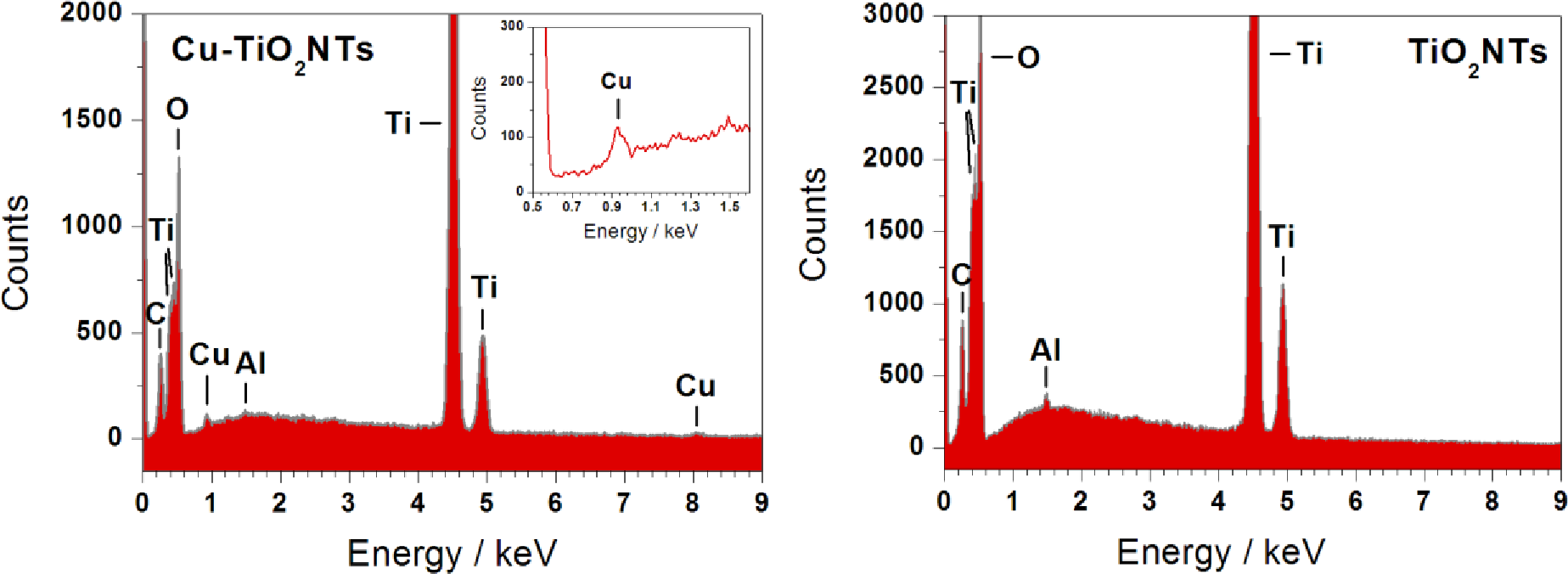

Supplement: S2 Fig — EDX spectra of copper doped TiO2 nanotubes (Cu-TiO2NTs, left) and B) undoped TiO2 nanotubes (TiO2NTs) (right). The inset in figure A unambiguously shows presence of copper in Cu-TiO2NTs. The amount of copper determined from the EDX spectrum of Cu-TiO2NTs is 1.2 wt%. In both spectra are present signals of C and Al what is a consequence of the specimen preparation for EDX analysis (please see Materials and Methods in the main paper). (TIF) [file pone.0227574.s002.tif]

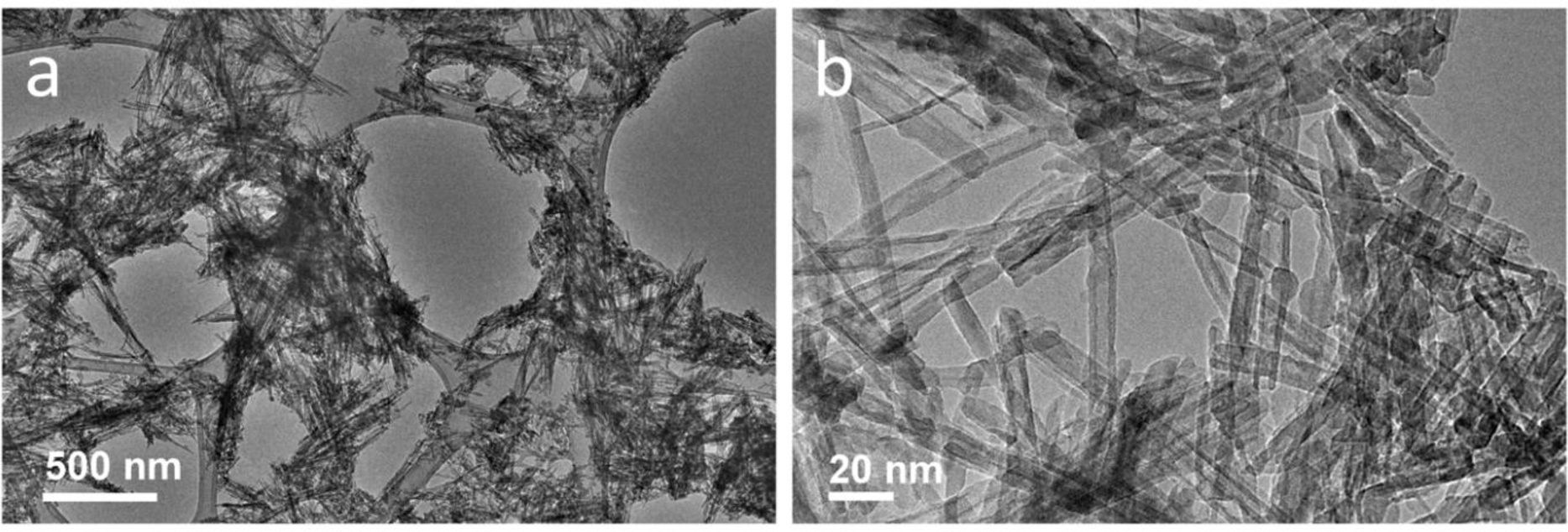

Supplement: S3 Fig — Low (a) and (b) high magnification TEM images of TiO2 nanotubes (TiO2NTs) obtained by calcination of H2Ti3O7 nanotubes at 380°C for 10 h. Nanotubes wall structure can be clearly seen in the inset of image (b). The average nanotube diameter is between 8 nm and 9 nm. (TIF) [file pone.0227574.s003.tif]

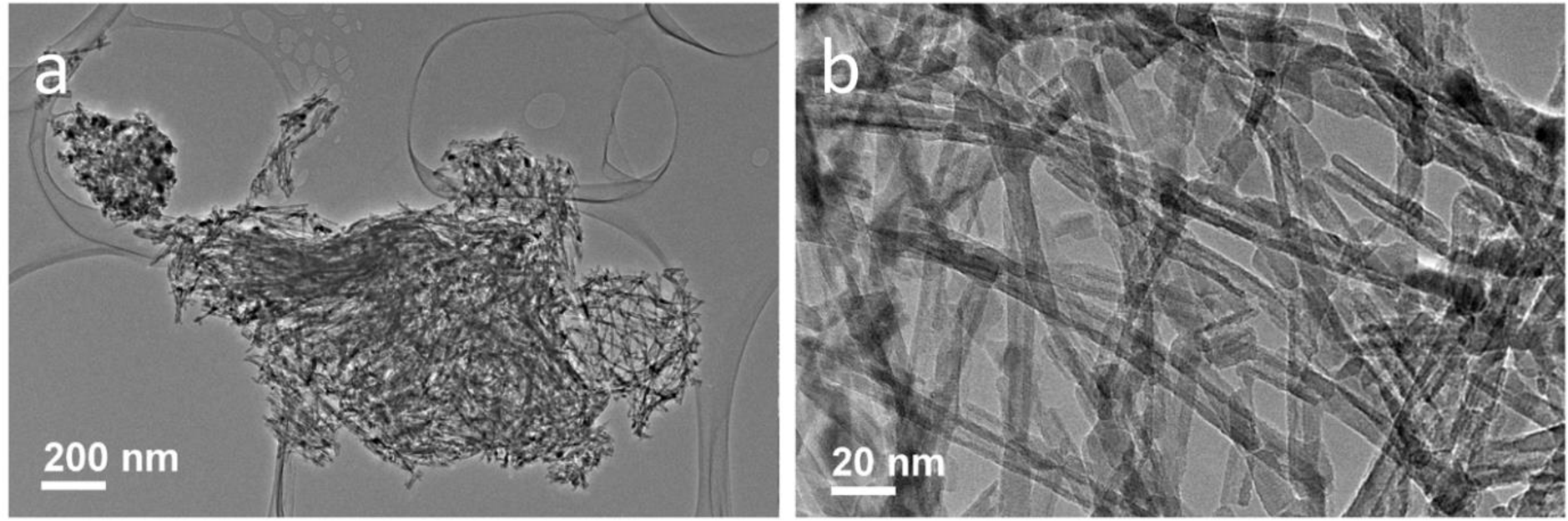

Supplement: S4 Fig — TEM images of copper doped TiO2 nanotubes (Cu-TiO2NTs) obtained by calcination of copper doped H2Ti3O7 nanotubes at 400°C at low (a) and (b) high magnification. Upon calcination nanotube morphology is retained. (TIF) [file pone.0227574.s004.tif]

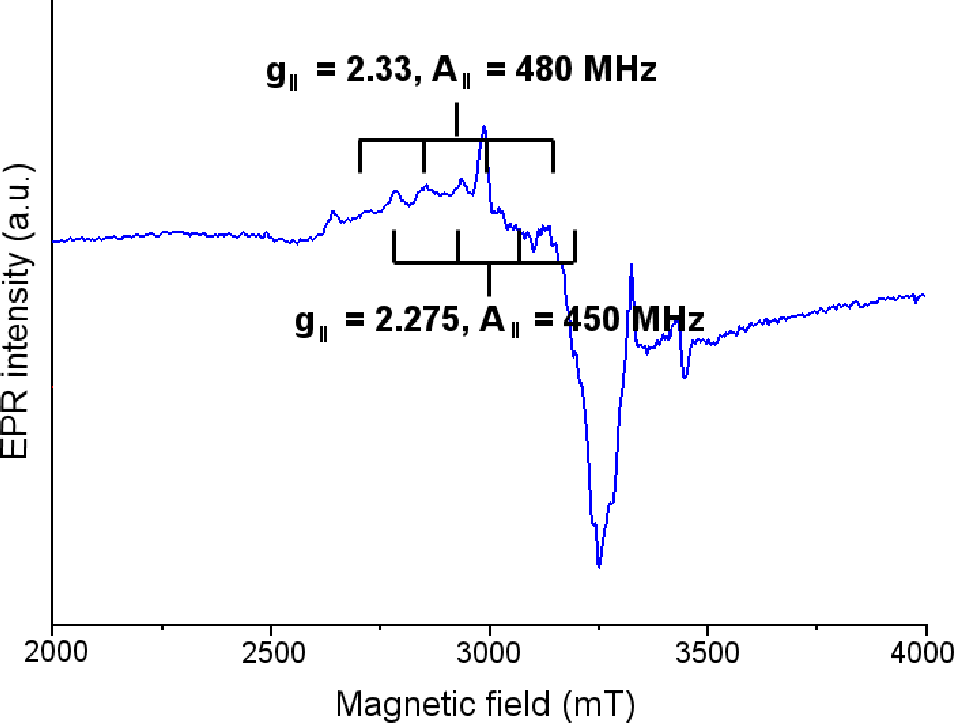

Supplement: S5 Fig — The black lines indicate hyperfine structure for two coexisting signals in Cu-TiO2NTs with 0.1 wt. % of copper. The EPR measurement was performed on an X-band EPR spectrometer Bruker ELEXYS, Type W3002180, using 10 Gauss (10−3 T) modulation amplitude, 100 kHz modulation frequency, 3 μW microwave power and 2000 G sweep. (TIF) [file pone.0227574.s005.tif]

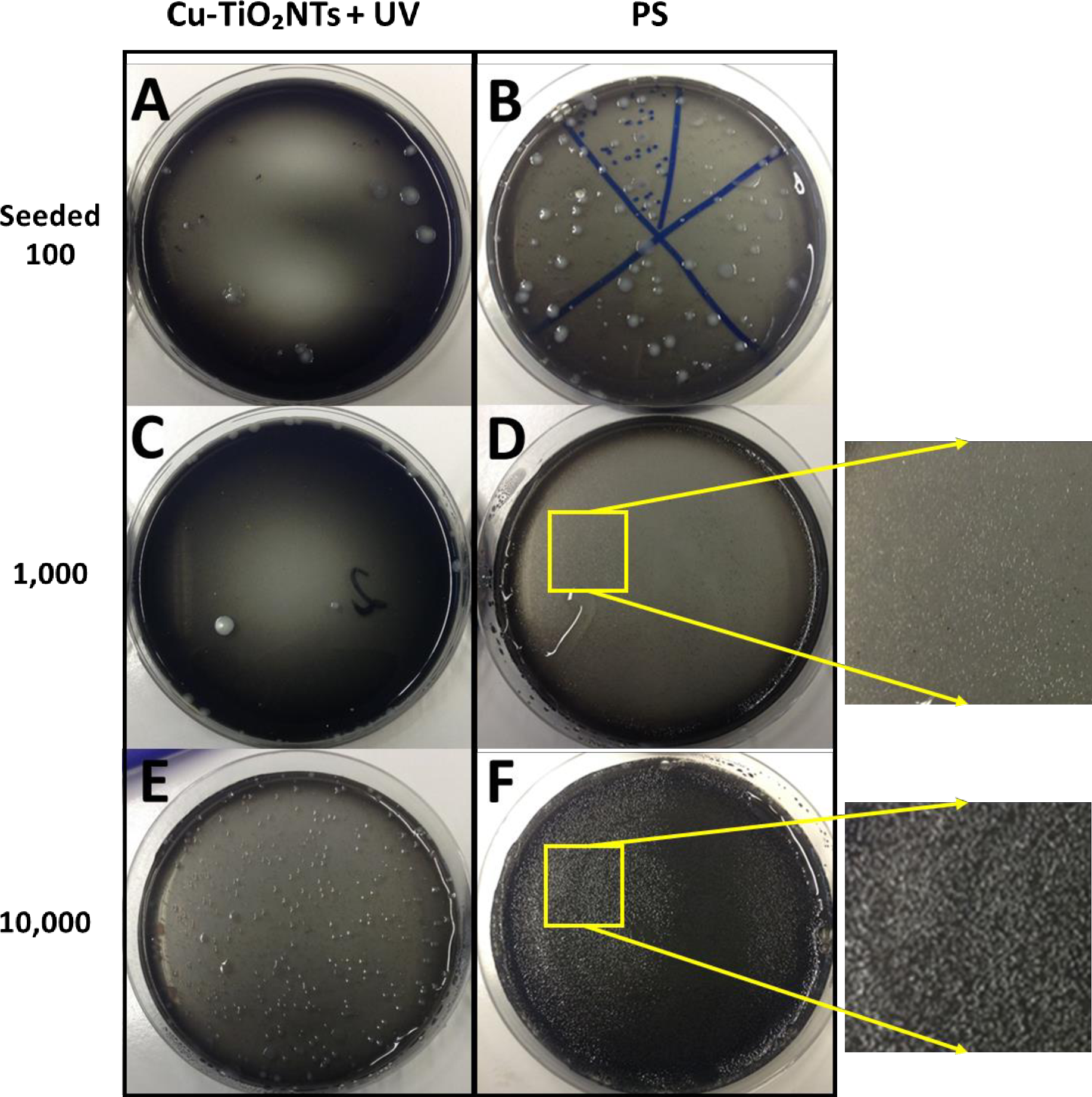

Supplement: S6 Fig — Survival of different number of Legionella pneumophila (100 bacteria seeded: A, B; 1,000 bacteria seeded: C, D; 10,000 bacteria seeded: E, F) in saline solution in a petri dish coated with copper doped TiO2 nanotubes (column Cu-TiO2NTs + UV) illuminated with UVA light (15 W/m2) incubated for 24 hours in incubator at 36˚ C compared to control (column PS–bare polystyrene surface). (TIF) [file pone.0227574.s006.tif]

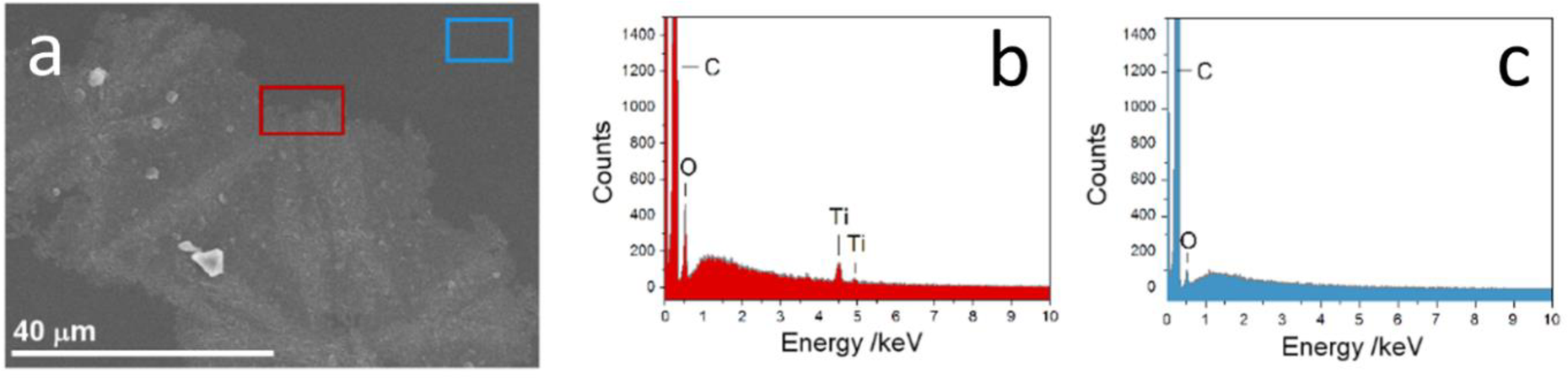

Supplement: S7 Fig — SEM image Cu-TiO2NTs deposited on a petri dish (a) and EDX spectra (b,c). Red frame in the SEM image corresponds to an area over which the spectrum (a) was taken, while blue frame corresponds to an area over which the spectrum (b) was taken. Spectrum (b) shows presence of Ti and O coming from TiO2, while in the spectrum (c) there are just signals of C and O. Presence of oxygen in this spectrum is probably a consequence of the cleaning process where compressed air treatment was used. (TIF) [file pone.0227574.s007.tif]

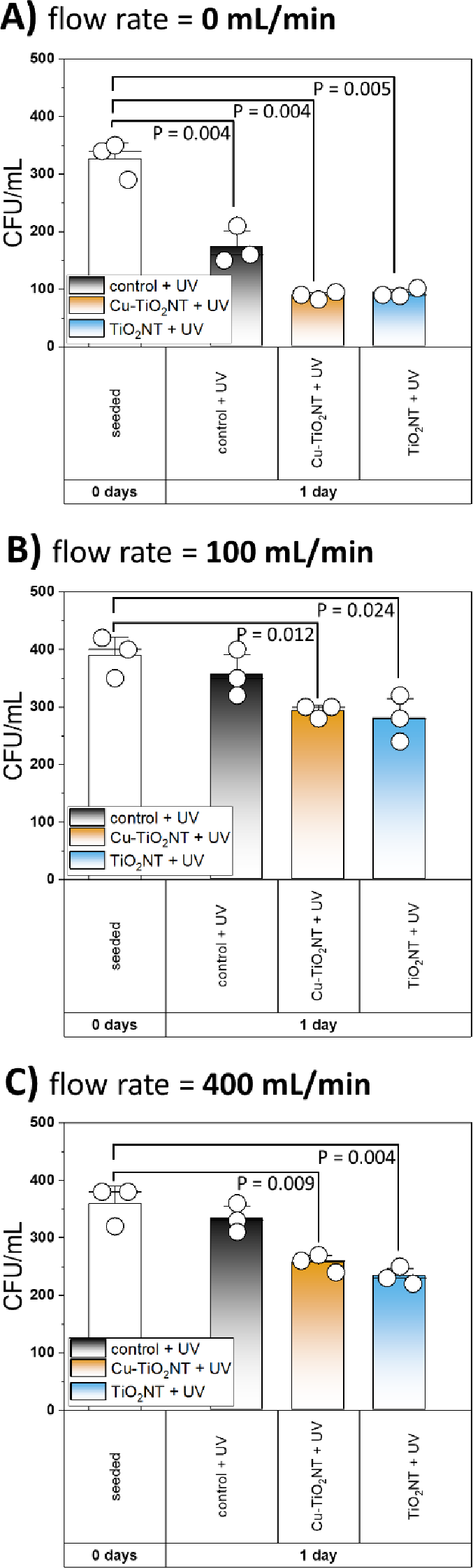

Supplement: S8 Fig — Volume of entire fluid was 2 liters. Legionella was grown overnight in suitable growth medium (overnight culture), which was diluted in a ratio of 1:30 into 2 liters of growth medium thus containing approximately 360 bacteria / mL (standard medium BCYE for Legionella with added yeast extract). Diluted bacteria were circulating in saline solution for 24 hours in a laminar flow chamber at 22°C. (TIF) [file pone.0227574.s008.tif]
