# Supplementary material for: Photocatalytic biocidal effect of copper doped TiO2 nanotube coated surfaces under laminar flow, illuminated with UVA light on Legionella pneumophila
Source: PLoS One. 2020 Jan 15;15(1):e0227574. doi: 10.1371/journal.pone.0227574 (PMC6961935; doi:10.1371/journal.pone.0227574)
Supplement: S1 Appendix — (DOCX) [file pone.0227574.s009.docx]

# **Statistical analysis of data shown in Figs 2 and 3**

Fig 2C:

**Paired t-test:**

**Data source:** Data 1 in Fig 2C statistical analysis - standing incubator 36C saline low UVA

**Normality Test (Shapiro-Wilk):**  Passed (P = 0.157)

**Treatment Name N Missing Mean Std Dev SEM**

seeded 3 0 250.000 132.288 76.376

3 days 3 0 120.000 72.111 41.633

Difference 3 0 130.000 60.828 35.119

t = 3.702 with 2 degrees of freedom.

95 percent two-tailed confidence interval for difference of means: -21.104 to 281.104

Two-tailed P-value = 0.0659

The change that occurred with the treatment is not great enough to exclude the possibility that the difference is due to chance (P = 0.066)

One-tailed P-value = 0.0329

# The sample mean of treatment seeded exceeds the sample mean of treatment 3 dayz by an amount that is greater than would be expected by chance, rejecting the hypothesis that the population mean of treatment 3 dayz is greater than or equal to the population mean of treatment seeded. (P = 0.066)

Fig 2D:

**t-test**

**Data source:** Data 1 in Fig 2D statistical analysis - standing incubator 36C saline high UVA

**Normality Test (Shapiro-Wilk):**  Passed (P = 0.270)

**Equal Variance Test (Brown-Forsythe):** Passed (P = 0.092)

The result of the equal variance test indicates the likelihood that the two groups are sampled from populations with equal variances, but does not guarantee the equality or inequality of the two variances.

**Group Name N Missing Mean Std Dev SEM**

seeded 4 0 142.500 71.356 35.678

1 day 4 0 15.500 11.705 5.852

Difference of means 127.000

Use the results of Welch's test, where equal variances are not assumed, if the equality of the population variances of the two groups is in doubt.

**Equal Variances Assumed (Student's t-test):**

t = 3.513 with 6 degrees of freedom.

95 percent two-tailed confidence interval for difference of means: 38.532 to 215.468

Two-tailed P-value = 0.0126

The difference in the mean values of the two groups is greater than would be expected by chance; there is a statistically significant difference between the input groups (P = 0.013).

**Equal Variances Not Assumed (Welch's t-test):**

t = 3.513 with 3.161 degrees of freedom.

95 percent two-tailed confidence interval for difference of means: 15.191 to 238.809

Two-tailed P-value = 0.0360

# The difference in the mean values of the two groups is greater than would be expected by chance; there is a statistically significant difference between the input groups (P = 0.036).

Fig 3A:

**t-test**

**Data source:** Data 1 in 2019 flow chamber

**Normality Test (Shapiro-Wilk):**  Passed (P = 0.726)

**Equal Variance Test (Brown-Forsythe):** Passed (P = 1.000)

The result of the equal variance test indicates the likelihood that the two groups are sampled from populations with equal variances, but does not guarantee the equality or inequality of the two variances.

**Group Name N Missing Mean Std Dev SEM**

seeded 3 0 326.667 32.146 18.559

uncoated 3 0 173.333 32.146 18.559

Difference of means 153.333

Use the results of Welch's test, where equal variances are not assumed, if the equality of the population variances of the two groups is in doubt.

**Equal Variances Assumed (Student's t-test):**

t = 5.842 with 4 degrees of freedom.

95 percent two-tailed confidence interval for difference of means: 80.461 to 226.206

Two-tailed P-value = 0.00428

The difference in the mean values of the two groups is greater than would be expected by chance; there is a statistically significant difference between the input groups (P = 0.004).

**Equal Variances Not Assumed (Welch's t-test):**

t = 5.842 with 4.000 degrees of freedom.

95 percent two-tailed confidence interval for difference of means: 80.461 to 226.206

Two-tailed P-value = 0.00428

The difference in the mean values of the two groups is greater than would be expected by chance; there is a statistically significant difference between the input groups (P = 0.004).

**Data source:** Data 1 in 2019 flow chamber

**Normality Test (Shapiro-Wilk):**  Passed (P = 0.529)

**Equal Variance Test (Brown-Forsythe):** Passed (P = 0.381)

The result of the equal variance test indicates the likelihood that the two groups are sampled from populations with equal variances, but does not guarantee the equality or inequality of the two variances.

**Group Name N Missing Mean Std Dev SEM**

seeded 3 0 326.667 32.146 18.559

TiO_2_NT coated 3 0 93.333 7.572 4.372

Difference of means 233.333

Use the results of Welch's test, where equal variances are not assumed, if the equality of the population variances of the two groups is in doubt.

**Equal Variances Assumed (Student's t-test):**

t = 12.237 with 4 degrees of freedom.

95 percent two-tailed confidence interval for difference of means: 180.394 to 286.272

Two-tailed P-value = 0.000256

The difference in the mean values of the two groups is greater than would be expected by chance; there is a statistically significant difference between the input groups (P = <0.001).

**Equal Variances Not Assumed (Welch's t-test):**

t = 12.237 with 2.221 degrees of freedom.

95 percent two-tailed confidence interval for difference of means: 151.294 to 315.373

Two-tailed P-value = 0.00441

The difference in the mean values of the two groups is greater than would be expected by chance; there is a statistically significant difference between the input groups (P = 0.004).

**Data source:** Data 1 in 2019 flow chamber

**Normality Test (Shapiro-Wilk):**  Passed (P = 0.575)

**Equal Variance Test (Brown-Forsythe):** Passed (P = 0.362)

The result of the equal variance test indicates the likelihood that the two groups are sampled from populations with equal variances, but does not guarantee the equality or inequality of the two variances.

**Group Name N Missing Mean Std Dev SEM**

seeded 3 0 326.667 32.146 18.559

Cu-TiO_2_NT coated 3 0 89.000 6.557 3.786

Difference of means 237.667

Use the results of Welch's test, where equal variances are not assumed, if the equality of the population variances of the two groups is in doubt.

**Equal Variances Assumed (Student's t-test):**

t = 12.547 with 4 degrees of freedom.

95 percent two-tailed confidence interval for difference of means: 185.077 to 290.257

Two-tailed P-value = 0.000232

The difference in the mean values of the two groups is greater than would be expected by chance; there is a statistically significant difference between the input groups (P = <0.001).

**Equal Variances Not Assumed (Welch's t-test):**

t = 12.547 with 2.166 degrees of freedom.

95 percent two-tailed confidence interval for difference of means: 156.168 to 319.165

Two-tailed P-value = 0.00462

The difference in the mean values of the two groups is greater than would be expected by chance; there is a statistically significant difference between the input groups (P = 0.005).

Fig 3B:

**Data source:** Data 1 in 2019 flow chamber

**Normality Test (Shapiro-Wilk):**  Passed (P = 0.334)

**Equal Variance Test (Brown-Forsythe):** Passed (P = 0.771)

The result of the equal variance test indicates the likelihood that the two groups are sampled from populations with equal variances, but does not guarantee the equality or inequality of the two variances.

**Group Name N Missing Mean Std Dev SEM**

seeded 3 0 390.000 36.056 20.817

TiO2NT coated 3 0 280.000 40.000 23.094

Difference of means 110.000

Use the results of Welch's test, where equal variances are not assumed, if the equality of the population variances of the two groups is in doubt.

**Equal Variances Assumed (Student's t-test):**

t = 3.538 with 4 degrees of freedom.

95 percent two-tailed confidence interval for difference of means: 23.677 to 196.323

Two-tailed P-value = 0.0241

The difference in the mean values of the two groups is greater than would be expected by chance; there is a statistically significant difference between the input groups (P = 0.024).

**Equal Variances Not Assumed (Welch's t-test):**

t = 3.538 with 3.958 degrees of freedom.

95 percent two-tailed confidence interval for difference of means: 23.311 to 196.689

Two-tailed P-value = 0.0245

The difference in the mean values of the two groups is greater than would be expected by chance; there is a statistically significant difference between the input groups (P = 0.024).

**Data source:** Data 1 in 2019 flow chamber

**Normality Test (Shapiro-Wilk):**  Passed (P = 0.559)

**Equal Variance Test (Brown-Forsythe):** Passed (P = 1.000)

The result of the equal variance test indicates the likelihood that the two groups are sampled from populations with equal variances, but does not guarantee the equality or inequality of the two variances.

**Group Name N Missing Mean Std Dev SEM**

seeded 3 0 390.000 36.056 20.817

Cu-TiO2NT coated 3 0 293.333 11.547 6.667

Difference of means 96.667

Use the results of Welch's test, where equal variances are not assumed, if the equality of the population variances of the two groups is in doubt.

**Equal Variances Assumed (Student's t-test):**

t = 4.422 with 4 degrees of freedom.

95 percent two-tailed confidence interval for difference of means: 35.979 to 157.355

Two-tailed P-value = 0.0115

The difference in the mean values of the two groups is greater than would be expected by chance; there is a statistically significant difference between the input groups (P = 0.011).

**Equal Variances Not Assumed (Welch's t-test):**

t = 4.422 with 2.406 degrees of freedom.

95 percent two-tailed confidence interval for difference of means: 2.619 to 190.715

Two-tailed P-value = 0.0335

The difference in the mean values of the two groups is greater than would be expected by chance; there is a statistically significant difference between the input groups (P = 0.034).

Fig 3C:

**Data source:** Data 1 in 2019 flow chamber

**Normality Test (Shapiro-Wilk):**  Passed (P = 0.195)

**Equal Variance Test (Brown-Forsythe):** Passed (P = 1.000)

The result of the equal variance test indicates the likelihood that the two groups are sampled from populations with equal variances, but does not guarantee the equality or inequality of the two variances.

**Group Name N Missing Mean Std Dev SEM**

seeded 3 0 360.000 34.641 20.000

TiO2NT coated 3 0 233.333 15.275 8.819

Difference of means 126.667

Use the results of Welch's test, where equal variances are not assumed, if the equality of the population variances of the two groups is in doubt.

**Equal Variances Assumed (Student's t-test):**

t = 5.795 with 4 degrees of freedom.

95 percent two-tailed confidence interval for difference of means: 65.979 to 187.355

Two-tailed P-value = 0.00441

The difference in the mean values of the two groups is greater than would be expected by chance; there is a statistically significant difference between the input groups (P = 0.004).

**Equal Variances Not Assumed (Welch's t-test):**

t = 5.795 with 2.749 degrees of freedom.

95 percent two-tailed confidence interval for difference of means: 32.619 to 220.715

Two-tailed P-value = 0.0130

The difference in the mean values of the two groups is greater than would be expected by chance; there is a statistically significant difference between the input groups (P = 0.013).

**Data source:** Data 1 in 2019 flow chamber

**Normality Test (Shapiro-Wilk):**  Passed (P = 0.202)

**Equal Variance Test (Brown-Forsythe):** Passed (P = 1.000)

The result of the equal variance test indicates the likelihood that the two groups are sampled from populations with equal variances, but does not guarantee the equality or inequality of the two variances.

**Group Name N Missing Mean Std Dev SEM**

seeded 3 0 360.000 34.641 20.000

Cu-TiO2NT coated 3 0 256.667 15.275 8.819

Difference of means 103.333

Use the results of Welch's test, where equal variances are not assumed, if the equality of the population variances of the two groups is in doubt.

**Equal Variances Assumed (Student's t-test):**

t = 4.727 with 4 degrees of freedom.

95 percent two-tailed confidence interval for difference of means: 42.645 to 164.021

Two-tailed P-value = 0.00912

The difference in the mean values of the two groups is greater than would be expected by chance; there is a statistically significant difference between the input groups (P = 0.009).

**Equal Variances Not Assumed (Welch's t-test):**

t = 4.727 with 2.749 degrees of freedom.

95 percent two-tailed confidence interval for difference of means: 9.285 to 197.381

Two-tailed P-value = 0.0218
